# Physiological and Productive Impacts of Including Artificial Saliva in Lamb Diets: Growth, Carcass Traits, and Fermentation Efficiency

**DOI:** 10.3390/vetsci13040395

**Published:** 2026-04-17

**Authors:** Mutassim M. Abdelrahman, Ibrahim A. Alhidary, Gamaleldin M. Suliman, Mohsen M. Alobre, Mohammed M. Qaid, Faisal A. Alshamiry, Abdulkareem M. Matar

**Affiliations:** Department of Animal Production, College of Food and Agricultural Sciences, King Saud University, P.O. Box 2460, Riyadh 11451, Saudi Arabia

**Keywords:** artificial saliva, blood parameters, performance, meat characteristics, rumen pH and color, Naemi lambs

## Abstract

The intensive lamb rearing and finishing systems are usually based on nutritionally balanced concentrate-based rations. However, the inclusion of feed additives is often necessary to avoid possible disturbances of digestibility. Artificial saliva plays an important role in ruminal buffering, improving the digestibility of the feed under conditions where natural saliva production may be insufficient. The study aimed to evaluate the effects of dietary artificial saliva (AS) on the total mixed ration (TMR) on lambs’ growth performance, carcass characteristics and the efficacy of ruminal fermentation. Forty-five male Naemi lambs (mean body weight of 23 ± 1.8 kg) were randomly assigned to five experimental groups (*n* = 9 per group) and kept in complete confinement with water and *ad* *libitum* feeding. During the 84-day feeding period, animals received isonitrogenous and isoenergetic TMRs that vary in AS inclusion by level: T0 (0% AS), T2 (1.5% AS), T3 (3.0% AS), T4 (4.5% AS), and T5 (6% AS). No significant differences (*p* > 0.05) were observed in the overall performance parameters. However, lambs fed the control diet (0% AS) had a higher body weight (BW), average daily gain (ADG), and feed intake (FI) than those receiving AS supplementation (*p* < 0.01). Ruminal pH measured 3 h after feeding and at slaughter remained comparatively stable in lambs fed 3% and 4.5% AS treatments, whereas it declined to 5.21 in the control group. Dietary inclusion of AS at 4.5% significantly increased (*p* < 0.05 < 0.05) the molar proportions of butyric acid, iso-valeric acid, and valeric acid in ruminal fluid while other levels of artificial saliva were associated with reduced concentrations of volatile fatty acids (VFAs). In conclusion, AS effectively functioned as a buffering agent, enhancing dietary safety without adverse health effects.

## Simple Summary

Artificial saliva is important to improving the rumen buffering capacity, digestibility of food and performance of the animals under conditions where natural saliva production may be insufficient. The study examined the effect of supplementing artificial saliva to the diet of lambs to improve their growth, digestion and carcass characteristics. Although the addition of the artificial saliva (AS) did not significantly alter the animals ‘ overall growth, lambs that did not receive it consumed more feed. However, lambs fed a moderate amount of artificial saliva (3 and 4.5%) maintained a healthier pH in the rumen, which helps to prevent problems with digestion. Overall, artificial saliva served as a natural buffer to stabilize the conditions of the rumen without causing any health problems when used with the 3% of AS.

## 1. Introduction

Rapid economic growth worldwide has increased the demand for animal-derived food products, driving the development of production systems that maximize biological and economic efficiency while keeping input costs under strict control. Livestock products and commodities represent a significant proportion of total consumer demand. In this context, the modern commercial sheep meat sector plays a key role in the efficient transformation resources from plant feed resources into high-quality animal proteins for human consumption [1].

Ruminant digestion is a highly complex, microbially mediated process that occurs primarily in the rumen, where fermentation of ingested substrates generates volatile fatty acids (VFAs) and microbial biomass that support the diet of the host [2]. The efficacy of the rumen fermentation depends on chemical and physical properties of the diet, the rate of salivary excretion, and physicochemical conditions of the rumen, such as pH, osmolarity, redox potential and microbial ecology [2].

The saliva is a critical modulator of the ruminal function. It provides a system of bicarbonate and phosphate supplementation to maintain the pH of the ruminal fluid within optimal physiological limits and provides the necessary electrolytes to support efficient microbial metabolism [3]. In conditions where endogenous salivary secretion is impaired or quantitatively deficient, such as during high-concentrate diets or under stress, supplementation with AS components has been proposed as an intervention to maintain ruminal homeostasis and enhance fermentation efficiency [4].

Previous studies have shown that inclusion of AS may affect rumen fermentation, nutrient degradation, microbial community in the rumen and physiological responses in ruminants [3,4,5]. Taken together, these observations highlight the need for a more comprehensive assessment of the AS in practical feeding systems, with particular emphasis on its effects on production performance. The profile of the metabolites of blood and meat characteristics that have not been characterized to a sufficient extent.

Nutritional management remains a major lever for improving sheep production systems, as feed accounts for more than 65% of total meat production of sheep [6]. Mineral supplementation strategies as buffering agents are therefore being evaluated to enhance nutrient uptake and growth performance [7]. Among buffering agents, sodium bicarbonate (NaHCO_3_) is widely used to mitigate ruminal acidosis, promote voluntary feed intake, increase body weight gain, and improve feed conversion efficiency [8].

Beyond its ruminal role, NaHCO_3_ contributes to systemic acid–base regulation. Under heat stress, increased respiratory activity enhances carbon dioxide (CO_2_) exhalation, altering acid–base dynamics and potentially challenging homeostasis [9]. Disturbances in acid–base balance can impair physiological function, tissue accretion, and overall performance [10]. Electrolyte balance, particularly dietary sodium (Na^+^), potassium (K^+^), and chloride (Cl^−^), is fundamental for maintaining acid–base equilibrium and fluid balance [11]. The acid–base balance reflects the organism’s capacity to maintain a relatively constant hydrogen ion concentration within both the intracellular and extracellular compartments, a process referred to as acid–base homeostasis. When this equilibrium is disturbed, particularly in the context of cellular metabolism, it can result in impairments such as decreased efficiency of tissue formation and reduced muscle accretion [12].

Artificial saliva, containing sodium bicarbonate, supplies Na^+^ and bicarbonate (HCO_3_^−^), which promote absorption capacity and may improve nutrient the digestibility and performance, particularly under thermal stress [11,13]. In addition, optimal electrolyte and acid–base conditions promote the growth of the metabolic environment rather than compensatory homeostatic adjustments, thus promoting body weight gain, feed intake, feed efficiency and quality of the carcass [14].

It was therefore hypothesized that dietary inclusion of AS would modulate ruminal fermentation, thereby enhancing growth performance and meat quality. Accordingly, this study evaluated the effects of incorporating AS at varying levels in total mixed rations on growth performance, ruminal fermentation efficiency, and carcass characteristics of Naemi lambs.

## 2. Materials and Methods

### 2.1. Study Location

The study was carried out between May and August 2023 at a small experimental farm belonging to the Animal Production Department, King Saud University, in Al-Ammariyah city (Riyadh Province, Saudi Arabia), situated at approximately 600 m above sea level. The ambient temperature during the trial period was 32 to 36 °C, and the natural photographic period was approximately 13.5 h/day.

### 2.2. Animals, Experimental Design, and Diets

At two weeks after weaning (75–85 days of age), forty-five male Naemi lambs were assigned to five dietary treatments using a randomized complete block design (RCBD), based on body weight (mean body weight 23 ± 1.8 kg). The animals were housed in 15 pens of 1.2 × 150 m^2^ (3 pens per treatment; 3 lambs per pen), and each pen was considered as an experimental unit, each equipped with a drinker and a feeder. Lambs were fed *ad libitum* for 84 days with five isonitrogenous and isoenergetic diets as the total mixed ration (TMR). The basal TMR consisted of barley (30.1%), palm kernel meal (18.5%), wheat bran (9.8%), alfalfa hay (19.5%), soya hulls (12.5%), salt (2.5%), limestone (3.5%), molasses (2.8%), and a commercial premix (0.08%: provided by Sustar compony; Shouan Town, Pujiang County, Chengdu City, Sichuan Province, China). The calculated chemical composition of the diets, expressed on a dry matter (DM) basis, was as follows: dry matter, 89.5%; crude protein (CP), 14.6%; crude fiber (CF), 12.95%; ether extract (EE), 2.50% and metabolizable energy (ME), 2.81 Mcal/kg.

The diets were supplemented with different levels of AS corresponding to the following treatments: control, T1, T2, T3, and T4, with AS inclusion levels of 0, 1.5, 3.0, 4.5, and 6.0%, respectively [5]. The AS product was provided as a powder mixture of sodium Bicarbonate, dipotassium Phosphate, monammonium phosphate and magnesium sulphate (manufacturing by David Taylor animal nutrition Ltd., Carrick Mill, Collinstown, Co. Weastmeath, Ireland). Analytical constituents were calcium 0.01%, sodium 17.19%, phosphorus 8.24%, and magnesium 0.09%. Water was available *ad libitum*. Daily feed consumption was recorded for each pen to estimate average intake per lamb within treatment.

### 2.3. Growth Performance

The amount of feed offered and refusals were recorded daily per pen to determine feed intake. Lambs were weighed weekly as a group, and average daily gain (ADG) was calculated as the difference between the final and initial live weight (LW) divided by 84 days. Daily feed intake (DFI) was determined by summing the total feed consumption per group during the trial period divided by 84 days and the number of lambs per group. The feed conversion ratio (FCR) was calculated as daily feed intake divided by ADG. All weighing procedures were performed using a calibrated electronic scale with a capacity of 100 kg and an accuracy of 0.01 g.

### 2.4. Carcass Traits

At the end of the study, six lambs per treatment were randomly selected and slaughtered by severing the carotid and jugular veins using a sharp knife in accordance with halal procedures, ensuring efficient exsanguination, minimizing pain, and promoting meat quality [15]. Hot carcass weight (HCW) and the pH of the carcass were recorded at 45 min post-mortem, while cold carcass (CCW) was determined after 24 h of chilling at 4 °C. Muscle and rumen pH were measured using a portable digital pH meter (Hanna Instruments Deutschland GmbH, Hanna^®^ HI 99163, Baden-Württemberg, Germany). The viscera were also removed from the carcass and weighed by the abovementioned scale. Carcass yield (CY, %) was calculated by dividing the weight of the HCW by 100, divided by the final weight [(HCW/final LW) × 100]. Drip loss (DL, %) was estimated from the difference between HCW and CCW. The gastrointestinal tract (GIT), liver, heart, lungs, perirenal fat, kidneys, rumen, and head were removed and weighed using Kern scale of 5000 g (±0.01 g). Organ weights were expressed as a percentage of empty body weight.

### 2.5. Rumen Fluid, Color, and pH

Ruminal fluid samples were collected from the same animals at 21-day intervals, at each sampling point both before and 3 h after feeding, using a stomach tube connected to a gentle vacuum pump in accordance with standard veterinary procedures. To minimize contamination of saliva, an initial 30 to 50 mL of ruminal fluid was discarded, and a subsequent aliquot was used for pH determination. The interval between sampling and pH measurement was kept under 2 min, and the measurements were conducted immediately in the field using a portable pH meter (Hanna^®^ HI 99163 Portable Digital pH Meter).

Samples were immediately frozen in liquid nitrogen and then stored at −20 °C for analysis. After melting at 4 °C, the samples were centrifuged at a rate of 14,000× *g* for 10 min. The resulting supernatant was used to determine the ammonium nitrate (NH_3_-N) and VFAs. The concentration of NH_3_-N was determined by the phenol–hypochlorite colorimetric method [13] using a spectrophotometer at 630 nm (Agilent Technologies, Cary 60 UV-vis- Santa Clara, CA 95051, United States). The VFA concentrations were analyzed by gas chromatography (Hewlett Packard Model HP 6890, Waldbronn, Germany) according to the standard procedures defined by Luo et al. [16].

Rumen samples from the dorsal cavity were extracted and used for chromatographic analysis. The color of the tissue was quantified using a Minolta Chromo-meter (Minolta, CR-400, Osaka, Japan) operating in the color space of the CIELAB, with the L-coordinate as the luminosity parameter.

### 2.6. Blood Serum Metabolites

Blood samples from each group were collected from the carotid artery and jugular veins of Naemi lambs. Samples were placed in heparinized tubes and centrifuged at 3500 rpm for 15 min at 5 °C [17]. The resulting serum was poured into two 1.5 mL Eppendorf and stored at −20 °C until analysis. Serum aspartate aminotransferase (AST) and alkaline phosphatase (APS) activity, total protein (TP), urea nitrogen (UN), and albumin content were determined using standard biochemical analyzers (Biobase BK-AA32ON atomic absorption spectrophotometer and the Yellow Springs YSI 2300 biochemical analyzer).

### 2.7. Statistical Analysis

Data on growth performance, blood metabolites, rumen fermentation, and carcass traits were analyzed using one-way ANOVA under Proc Mixed procedure of SAS (Version 9.4, SAS Institute Inc., Cary, NC, USA). The model included dietary AS level as a fixed effect:

Y_ij_ = μ + α_i_ + ϵ_ij_

where

* Y_ij_ = observation value for the j-th observation in the i-th group.

* μ = overall mean of all observations.

* α_i_ = effect of the i-th group (treatment).

* ϵ_ij_ = random error term for the j-th observation in the i-th group

The statistical models included different levels of AS in diets as main effects. Duncan’s test was used to detect differences between treatment means, and statistical significance was declared at a *p* < 0.05 level. The impact of the levels of AS dose was evaluated utilizing orthogonal polynomial contrasts, specifically through linear and quadratic adjustments.

## 3. Results

### 3.1. Growth Performance

The mean values for body weight (BW), body weight gain (BWG), average daily gain (ADG), feed intake (FI), and feed conversion ratio (FCR) of the Naemi lambs are presented in Table 1. Performance results indicated significant differences (*p* < 0.05) between the final BW, BWG, ADG and FI between lambs fed a control diet and those receiving AS supplementation. Lambs fed the control diet exhibited greater performance indices compared with the AS-supplemented groups. However, when evaluating performance during the intermediate (45 days) and overall (84 days) experimental periods, AS supplementation did not consistently improve BW, BWG, ADG, or FI relative to the control (Table 1). Polynomial contrasts indicated the performance parameters declined significantly compared with 0% for most variables in a linear fashion. Only BW had a quadratic effect, and it declined then leveled off/bottomed out.

The metabolic blood parameters are presented in Table 2. Serum glucose concentrations were significantly affected (*p* < 0.05) by dietary AS supplementation at both 42 and 84 days. Lambs receiving the control diet and the 6% AS treatment showed significantly different glucose levels compared with the intermediate inclusion levels. Increasing AS supplementation from 1.5% to 6% was associated with a progressive rise in serum glucose concentration. Serum total protein increased linearly (*p* < 0.05) with increasing dietary AS level throughout the study period. Conversely, serum albumin exhibited the highest concentration at 1.5% AS inclusion and declined gradually as, AS level increased to 6%. Serum cholesterol displayed a significant quadratic response (*p* < 0.05) to AS supplementation, increasing up to 3% inclusion level and subsequently decreasing at higher levels, with the lowest concentration observed at 6% AS. Serum urea concentration was also significantly influenced by dietary treatment (*p* < 0.05), with the highest value observed at 3% AS and the lowest at 1.5%.

### 3.2. Meat Traits

#### 3.2.1. Carcass Composition and Body Components

Table 3 shows the effects of dietary AS levels on carcass characteristics. Supplementation with AS at inclusion levels ranging from 1.5% to 6% did not significantly influence carcass weight, major carcass components, or overall body composition. However, tail weight and full stomach weight were significantly affected (*p* < 0.05), particularly in lambs receiving the 3% AS treatment. Furthermore, empty stomach weight was significantly greater in the 6% AS group compared with the other groups.

#### 3.2.2. Fat Depots and Primal Wholesale Cuts

Dietary AS supplementation did not significantly alter most fat depots or the primary wholesale cuts. Exceptions were observed for KKCF (kidney, knob, and channel fat), which was significantly affected at the 1.5% inclusion level, whereas the fore shank and breast cut showed significant differences at the 3% AS levels as shown at (Table 4).

### 3.3. Rumen Color and pH

**Figure 1:**
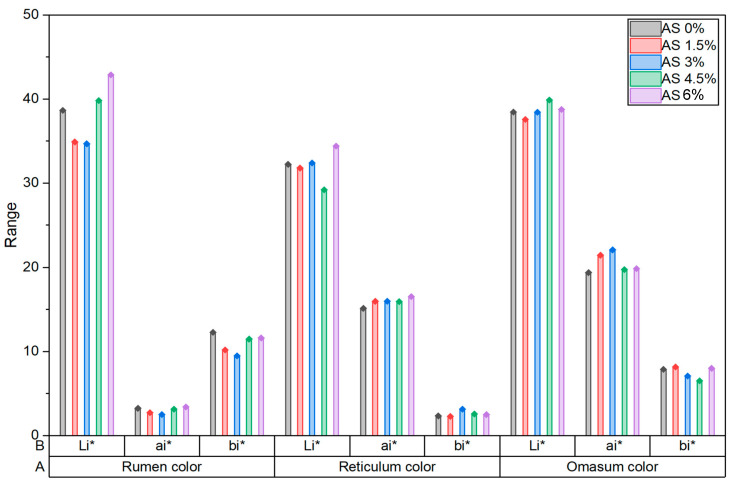
Influence of feeding total mixed ration (TMR) with different levels of artificial saliva (AS) on the rumen color, reticulum color, and omasum color in Naemi lambs.

Figure 1 illustrates the effect of increasing dietary AS inclusion levels on rumen, reticulum and omasum color parameters. AS supplementation significantly influenced lightness (L*) of the rumen and the redness (a*) of the omasum (*p* < 0.05), whereas the remaining color indices were not markedly affected.

**Figure 2:**
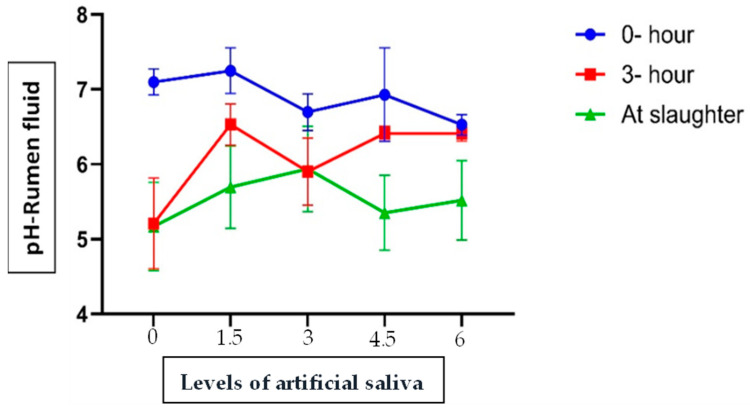
Effect of the total mixed ration (TMR) with different levels of artificial saliva (AS) on the rumen pH at 0 and 3 h after feeding and at slaughter in Naemi lambs.

Ruminal pH dynamics are presented in Figure 2. Measurements were taken before feeding, 3 h post-feeding, and after slaughter. Three hours after feeding, lambs receiving AS supplementation maintained relatively higher and more stable ruminal pH values (6.53, 5.94, 6.40, and 5.42 for 1.5%, 3.0%, 4.5%, and 6.0% AS, respectively), compared with the control group, in which pH declined to 5.21. A similar trend was observed at slaughter, where ruminal pH values in AS-treated groups (5.70, 5.92, 5.35, and 5.52) were consistently higher than that of the control group (5.17).

Table 5 summarizes the performance of rumen fermentation and the effect of different levels of AS in the diet on the concentration of ammonia nitrogen (NH_3_-N) and volatile fatty acids (VFA) in the rumen fluid of Naemi lambs.

Data shows that 4.5% of AS included in the diet significantly increased the ratios of minor VFAs (butyric acid, iso-valeric acid, and valeric acid). On the other hand, the main VFAs, including acetic and propionic acids, and ammonia were not significantly affected by the addition of AS.

This was followed by the 3% AS treatment, while the other treatments exhibited relatively lower concentrations of these volatile fatty acids (VFAs).

## 4. Discussion

### 4.1. Growth Performance

The digestive process in ruminants such as lambs involves complex interactions of microbial fermentation in the lining of the rumen, a specialized area of the stomach. Ruminant production systems often adopt strategies to increase feed efficiency and animal welfare, with particular emphasis on modulation of complex biochemical and microbiological processes in the rumen [18]. One such strategy involves the incorporation of AS in feedstuffs to optimize ruminal fermentation and nutrients assimilation [3]. The use of sodium bicarbonate reproduces the beneficial properties of natural saliva, which are essential to replenish and regulate the pH of the ruminal fluid, to provide it with essential mineral ions and to support the growth and metabolic activity of the ruminal microorganism [19].

Artificial saliva helps to maintain a stable ruminal environment and may increase the efficiency of feed conversion and the growth performance of lambs [20]. In particular, optimal incorporation of AS will improve the growth performance, hematological, biochemical blood parameters, nutritional tolerance, and VFA concentration of the growing lambs [21]. Previous studies have investigated the effects of AS on the bacterial fermentation patterns and community structure in in vitro systems and demonstrated that it is important for the regulation of microbial activity and metabolite formation [3]. However, further in vivo studies are needed to elucidate these effects under practical feeding conditions in lambs, with specific digestive and growth characteristics [3].

The findings of this study are interpreted in the light of the existing literature to elucidate the effects the supplementation of AS on lamb performance, ruminal function, and meat quality. A detailed understanding of the interaction between AS and the digestive system of lambs is essential to assess its impact on growth performance. This requires an assessment of key production and nutritional parameters, including DMI, ADG, FCR, and nutrient digestibility coefficients. Each of these indicators contributes to the determination of the efficacy of the AS in supporting optimum growth and health in lambs.

In this study, the inclusion of AS at different levels in the lamb feed did not result in significant differences in growth performance compared to the control group. These results indicate that the supplementation AS did not increase the productive response under current feeding conditions. These findings indicate that the basal total mixed ration provided adequate ruminal buffering capacity and that additional AS may have reduced the intake of feed or altered the fermentation dynamics in a way that would not have promoted better growth results. These results are in agreement with the findings of Mahdavirad et al. [22] in Arabi sheep, where no significant differences were reported among experimental treatments for initial and final body weights, ADG, FCR, DMI, or blood parameters. Consistent findings have also been reported in Kashmir lambs, in which supplementation with sodium bicarbonate did not significantly affect DMI, ADG, FCR, or nutrient digestibility coefficients [23]. Similarly, Abdelrahman et al. [5] reported the supplementation of AS had not affected growth performance.

On the other hand, other studies have shown that the use of AS enhances growth performance of the Awassi lambs, where the inclusion of 0.4% acid buffer in the diet, in combination with NaHCO_3_ (Buf2), improved FCR compared to the control group [21]. The improvement in feed efficiency was accompanied with alterations in the characteristics of the ruminal fermentation, notably a decrease in propionic acid concentration and an increase in butyric acid concentration [21]. Similarly, the inclusion of AS into concentrate-based diets at an ambient temperature of 20 °C has been shown to increased FI and ADG, thereby improving overall growth performance in lambs [24].

### 4.2. Meat Traits

In current studies, the supplementation of AS in lamb diets has shown no significant effects on major meat traits such as carcass weight, dressing percentage, or general meat quality parameters. However, AS inclusion has affected significant fat tail, KKC, fore shank and breast traits.

Studies in Merino sheep have demonstrated that the inclusion of 2% NaHCO_3_ in the concentrate diet may increase the quality of the meat, in particular in terms of color and tenderness [25]. Similarly, in lambs raised at high ambient temperatures, dietary supplementation with NaHCO_3_ was associated with improved color of the meat, increased water holding capacity, and favorable shear force, indicating a higher overall quality of the meat [24]. Conversely, other studies have shown that supplementation of NaHCO_3_ in concentrate diets has minimal influence on characteristics of carcass and quality of the meat, but the dressing yield has improved and the total carcass of fat was reduced when a buffer is added [26].

In addition, Vicente et al. [27] reported that supplementation with 20 g/kg of buffer resulted in a quadratic increase in final and slaughter weight, resulting in a significant increase for the loin muscle area, leg compactness index, weights of neck, shoulder, rib, and leg cuts in the hot and cold carcass weights. However, its specific effects on the characteristics of lamb carcass remain unclear and not yet fully understood [28].

### 4.3. Rumen Color and pH

In the first weeks of life, lambs have relatively low pH in the rumen (~5.2–5.5), mainly because the esophageal passage leads milk to the abomasum, but some milk can enter the rumen, where it ferments rapidly thus promoting an acid ruminal environment [29]. Moreover, the production of saliva is limited at this stage as the lambs are not yet chewing and digesting their feed, which limits the supply of endogenous buffers that can increase the pH of the ruminal fluid [30]. After weaning, lambs become increasingly dependent on feed as their primary source of nutrition, and the physiological and microbial mechanisms involved in fiber metabolism become fully expressed. In these conditions, the rumen pH usually exceeds 6.2, which is considered optimal for cellulolytic bacteria proliferation and activity [30]. Artificial saliva contain sodium bicarbonate, which mimics the buffering capacity of natural saliva, enhancing the neutralization of acidity of the ruminal, thus contributing to a more stable pH of the rumen [27]. The stabilizing of the pH of the ruminal acid creates a more favorable environment for the resident microbial community, which is essential to maintain the efficient fermentation and degradation of dietary substrates. Under well-buffered conditions, the digestion of fiber fractions is improved, leading to better nutrient availability, absorption, and overall metabolic efficiency of the lambs. Therefore, improved ruminal pH regulation and improved digestive efficiency associated with the intake of sodium bicarbonate may result in improved health, weight gain and overall improved growth performance [31]

In this study, supplementary AS improved and stabilized the pH of the rumen, especially 3 h after feeding. This effect is due to the provision of sodium and bicarbonate ions, which support the rebalancing of electrolytes and the maintenance of pH homeostasis in the stomach. In addition, AS supplementation improved the fluid clarity of the rumen (L*) and redness of the omental tissue (a*) compared to the control group. Color parameters (L*, a*, b*) are often used as indirect indicators of gastrointestinal tissue condition, as they may reflect factors such as integrity of the mucosa, vascularization, hydration status, or the mild inflammatory response. Therefore, slight alterations in L* can be associated with changes in tissue brightness or surface hydration, whereas variations in a* may indicate shifts in tissue oxygenation or blood flow. However, these measurements do not directly correspond to commercial meat quality attributes and should be interpreted primarily from a physiological perspective. Accordingly, these findings should be regarded as subtle indicators of tissue response rather than strong signs of physiological changes induced by treatment.

In the feed of lambs, the use of AS is particularly beneficial in intensive finishing systems based on a high fat and high protein diet acts to avoid excessive ruminal acidification, thus maintaining the microbial ecosystem of the ruminal gland and promoting efficient breakdown of both fiber and starch components of the diet [27]. A study in Arab sheep has shown that dietary supplementation with sodium bicarbonate increases the pH of the rumen and decreases the ammonia–nitrogen concentration, which suggests an increase in the efficiency of ruminal fermentation [22]. Although this method is effective in maintaining the pH of the rumen, it may be associated with a reduced digestibility of the fiber relative to other ingredients, with possible implications for the productivity of the animal and the overall fermentation efficiency.

Abdelrahman et al. [5] reported that AS supplementation improves the color of the rumen and contributes to maintaining the pH of the rumen in lambs, further supporting the role of buffering agents in maintaining the health and function of the rumen. In addition, evidence from in vitro and in vivo studies suggests that the addition of NaHCO_3_ to lamb diets increases the rumen pH and VFA concentrations, while reducing the concentrations of lactic acid and several biogenic amines (methylamine, tryptamine, tyramine, histamine, and putrescine) [19].

In the current study the concentrates of some VFA increased with the supplement of AS, such as butyric, iso-valeric, and valeric acids. These results were in agreement with the study by [19,32] which reported the increase in pH is accompanied by changes in the VFAs profile: concentrations of acetate, propionate, iso-butyrate, isovalerate and valerate are increased while butyrate concentrations are decreased.

This is supported by a physiological study showing that specialized bicarbonates (e.g., AE2, DRA, PAT1) are required for the transport of bicarbonate from the intracellular compartment of the epithelial cells of the ruminant rumen lumen are essential for VFA absorption [33]. Luminal bicarbonate secretion buffers H^+^, thereby preventing a pH decline in rumen and promoting homeostasis of intracellular acid-dependent carbonic anhydrase in epithelial cells. In addition, this process maintains a favorable pH gradient in the trans-epithelial that preserves VFAs in their readily absorbable, undissociated form and prevents intracellular acidification, allowing for a durable absorption of VFA [33].

As stated by Mao et al. [19], the changes in pH and VFA composition create a more favorable environment for cellulolytic microorganisms, as reflected by an increased variety of bacteria, the increased relative abundance of genera that degrade fibers, such as *Ruminococcus* and *Prevotella*, and the reduction in the proportion of less beneficial taxa such as *Streptococcus* and *butyrivibrio*. Thus, stabilization of the pH of the rumen enhances the degradation of the fiber feed, improves microbial protein synthesis and enhances the efficiency of nitrogen use [32]. Finally, AS acts as a buffer to reduce ruminal acidosis, promotes a more stable and functionally efficient microbial ecosystem in the ruminal tract, and promotes improved fiber digestion, thus contributing to improved lambs’ health.

## 5. Conclusions

The results indicate that under the conditions of the present study, dietary incorporation of AS did not affect significantly on performance or meat quality in Naemi lambs. Nevertheless, it has been observed that inclusion levels of 3.0% and 4.5% AS appear to support more favorable ruminal pH stability and improved rumen color lightness (L*). These responses suggest a potential modulatory effect on ruminal fermentation, even though such effects were not consistently reflected in growth performance metric. It should be emphasized that optimal AS inclusion rates may depend on diet composition, management practices, and environmental conditions, including thermal stress. Therefore, further controlled studies under different production scenarios and conditions of digestibility, ruminal microbial and protein synthesis are warranted to determine the most appropriate supplementation strategies for different lamb production systems.

## Figures and Tables

**Table 1:** Effect of feeding total mixed ration (TMR) with different levels of artificial saliva (AS) on growth performance, feed intake (FI), and feed conversion ratio (FCR) of the Naemi lambs.

Parameters	Artificial Saliva (%)					SEM	*p Value*		
	0	1.5	3.0	4.5	6.0		Treat	Linear	Quadratic
Body weight, (kg)									
1 d	23.62	23.62	23.62	23.62	23.60	0.23	1.00	0.96	0.95
1–42 d	38.54 ^a^	36.27 ^ab^	36.09 ^b^	34.46 ^c^	34.67 ^bc^	0.77	0.02	0.002	0.19
1–84 d	51.78 ^a^	47.38 ^b^	48.06 ^ab^	46.78 ^bc^	46.62 ^c^	0.85	0.01	0.004	0.02
Body weight gain, (g/d)									
1–42 d	14.92 ^a^	12.64 ^b^	12.47 ^b^	10.83 ^c^	11.07 ^bc^	0.71	0.01	0.001	0.16
43–84 d	13.23	11.11	11.96	12.32	11.95	0.65	0.31	0.53	0.25
1–84 d	28.16 ^a^	23.76 ^bc^	24.43 ^b^	23.16 ^c^	23.02 ^c^	0.78	0.005	<0.0001	0.005
Average daily gain, (g/d)									
1–42 d	0.36 ^a^	0.30 ^b^	0.30 ^b^	0.26 ^c^	0.26 ^c^	0.02	0.01	0.001	0.19
43–84 d	0.32	0.27	0.29	0.30	0.29	0.02	0.31	0.47	0.27
1-84 d	0.34 ^a^	0.28 ^b^	0.29 ^ab^	0.28 ^b^	0.27 ^c^	0.01	0.005	0.001	0.006
Feed intake, (kg/d)									
1–42 d	1.43	1.41	1.33	1.34	1.32	0.05	0.51	0.14	0.68
43–84 d	1.62 ^a^	1.42 ^bc^	1.51 ^ab^	1.49 ^b^	1.19 ^c^	0.07	0.02	0.01	0.28
1–84 d	1.53 ^a^	1.42 ^b^	1.42 ^b^	1.41 ^bc^	1.26 ^c^	0.05	0.07	0.01	0.61
Feed conversion ratio, (kg FI/kg BWG)									
1–42 d	4.03	4.73	4.48	5.24	5.05	0.28	0.07	0.01	0.39
43–84 d	5.02	5.26	5.20	4.99	4.16	0.34	0.23	0.14	0.15
1–84 d	4.55	5.02	4.89	5.13	4.61	0.20	0.27	0.73	0.06

^a–c^ Means with different superscripts within each row were significantly different for five treatments at *p* < 0.05; SEM = standard error of mean; *p* = probability level.

**Table 2:** Effect of feeding total mixed ration (TMR) with different levels of artificial saliva (AS) on serum metabolites Naemi lambs.

Parameters	Artificial Saliva (%)					SEM	*p* Value		
	0	1.5	3.0	4.5	6.0		Treat	Linear	Quadratic
Glucose, g/L									
1 d	64.07 ^ab^	77.80 ^a^	56.12 ^bc^	58.32 ^b^	53.72 ^c^	3.87	0.01	0.01	0.38
42 d	60.82 ^a^	45.42 ^c^	52.95 ^b^	50.56 ^bc^	58.56 ^ab^	3.22	0.03	0.95	0.02
84 d	53.48 ^a^	48.07 ^b^	45.10 c	52.61 ^ab^	53.53 ^a^	1.84	0.02	0.47	0.01
Total Protein, g/L									
1 d	5.29 ^bc^	5.23 ^c^	5.37 ^b^	5.72 ^ab^	6.10 ^a^	0.26	0.17	0.02	0.25
42 d	5.08 ^c^	5.31 ^b^	5.22 ^bc^	5.66 ^ab^	5.87 ^a^	0.25	0.22	0.05	0.64
84 d	5.74	6.10	5.94	5.75	5.74	0.33	0.92	0.75	0.56
Albumin, g/L									
1 d	2.24	1.88	2.37	1.87	3.09	0.31	0.08	0.13	0.11
42 d	1.76	2.00	1.79	1.96	1.83	0.11	0.46	0.78	0.40
84 d	1.35 ^c^	1.79 ^a^	1.63 ^b^	1.64 ^b^	1.32 ^c^	0.13	0.10	0.64	0.02
Globulin, g/L									
1 d	3.02	3.32	3.00	3.85	3.20	0.30	0.32	0.35	0.53
42 d	3.33	3.31	3.44	3.70	4.04	0.28	0.34	0.07	0.45
84 d	4.40	4.31	4.31	4.11	4.43	0.35	0.96	0.90	0.67
Cholesterol, g/L									
1 d	68.50 ^ab^	60.97 ^c^	53.75 ^c^	67.16 ^b^	74.11 ^a^	4.09	0.03	0.07	0.01
42 d	73.17	66.83	72.27	71.73	79.45	7.23	0.81	0.50	0.39
84 d	60.88 ^c^	62.48 ^b^	82.08 ^a^	67.27 ^ab^	62.09 ^b^	3.03	0.01	0.62	0.01
Triglyceride, g/L									
1 d	71.91	65.08	87.30	66.13	74.73	5.70	0.11	0.82	0.71
42 d	71.90	67.93	65.43	69.38	69.73	6.08	0.96	0.96	0.59
84 d	65.48	81.20	57.30	62.50	68.50	5.05	0.06	0.36	0.71
Serum Urea, g/L									
1 d	15.37 ^b^	9.18 ^c^	20.13 ^a^	16.63 ^b^	20.58 ^a^	1.43	0.01	0.01	0.15
42 d	8.95	8.45	12.25	9.75	14.95	2.49	0.36	0.14	0.62
84 d	14.23 ^b^	9.88 ^c^	20.28 ^a^	16.15 ^ab^	14.95 ^b^	1.84	0.02	0.21	0.25

^a, b, and c^ Means with different superscripts within each row were significantly different for five treatments at *p* ≤ 0.05; SEM = standard error of the mean; *p* = probability level.

**Table 3:** Effect of feeding total mixed ration (TMR) with different levels of artificial saliva (AS) on carcass composition and body components in Naemi lambs.

Parameters	Artificial Saliva (%)					SEM	*p* Value		
	0	1.5	3.0	4.5	6.0		Treat	Linear	Quadratic
* Carcass composition:									
Slaughter wt., kg	51.78 ^a^	47.38 ^b^	48.06 ^ab^	46.78 ^bc^	46.62 ^c^	0.85	0.01	0.004	0.02
Hot carcass, kg	23.46	23.04	24.05	22.74	22.03	1.41	0.88	0.22	0.31
Cold carcass, kg	23.03	22.47	23.68	22.31	21.50	1.43	0.90	0.20	0.28
* Dressing, %	45.37	48.33	50.01	48.66	47.50	3.04	0.85	0.67	0.15
Chill shrink, %	1.85	2.55	1.60	1.92	2.42	0.37	0.39	0.30	0.31
* Body components (%):									
Head	6.60	6.74	6.58	6.85	7.13	0.30	0.76	0.06	0.17
Heart	0.75	0.80	0.73	0.72	0.76	0.03	0.51	0.18	0.24
Lungs	2.07	2.25	1.92	2.02	2.10	0.13	0.49	0.72	0.32
Liver	3.45	3.43	3.41	3.35	2.88	0.17	0.23	0.17	0.72
Spleen	0.34	0.32	0.31	0.36	0.39	0.03	0.37	0.23	0.32
Kidneys	0.55	0.56	0.66	0.67	0.63	0.05	0.19	0.42	0.61
Tail	14.33 ^ab^	11.09 ^c^	16.21 ^a^	13.32 ^bc^	13.47 ^b^	1.76	0.01	0.13	0.09
Stomach full	4.56 ^ab^	4.41 ^b^	4.79 ^a^	4.09 ^bc^	3.88 ^c^	0.41	0.04	0.02	0.07
Stomach empty	1.55 ^bc^	1.50 ^bc^	1.58 ^b^	1.45 ^c^	2.06 ^a^	0.12	0.01	0.09	0.04

^* a, b, and c^ Means with different superscripts within each row were significantly different at *p* < 0.05; SEM = Standard error of mean; *p* = Probability level. * body components (head, heart, lungs, liver, spleen, kidneys, tail, stomach full and stomach empty) is calculated by divided on weight of the carcass by 100.

**Table 4:** Effect of feeding total mixed ration (TMR) with different levels of artificial saliva (AS) on fat depots and primal wholesale cuts in Naemi lambs.

Parameters	Artificial Saliva (%)					SEM	*p* Value		
	0	1.5	3.0	4.5	6.0		Treat	Linear	Quadratic
* Fat Depots:									
* KKCF	1.35 ^ab^	1.66 ^a^	0.96 ^bc^	1.23 ^b^	0.76 ^c^	0.14	0.01	0.69	0.39
* Pericardial fat	0.35	0.39	0.32	0.40	0.28	0.03	0.18	0.77	0.89
* Omental fat	0.57	0.63	0.46	1.93	0.20	0.55	0.29	0.84	0.37
Backfat: mm	7.34	6.79	10.79	9.27	3.20	2.81	0.42	0.40	0.06
Body wall fat: mm	8.72	7.24	9.49	7.69	5.00	1.81	0.49	0.25	0.37
**Primal Wholesale Cuts (%):									
Shoulder	29.76	28.99	30.62	30.03	31.15	1.03	0.64	0.27	0.69
Rack	9.93	10.56	10.19	10.60	9.94	1.13	0.98	0.98	0.68
Loin	13.16	13.74	11.80	12.70	12.54	0.79	0.52	0.39	0.65
Leg	30.02	30.74	30.09	29.93	21.42	0.95	0.77	0.46	0.52
Fore shank and breast	17.13 ^ab^	15.98 ^bc^	17.30 ^a^	16.75 ^b^	14.96 ^c^	0.49	0.02	0.05	0.13

* Fat depots were calculated based on hot carcass without tail. ** Cuts were calculated based on hot carcass without tail; KKCF: kidney, knob, and channel fat; SEM = Standard error of mean; ^a, b, and c^ means with different superscripts within each row were significantly different at probability level *p* < 0.05.

**Table 5:** Effect of feeding total mixed ration (TMR) with different levels of artificial saliva (AS) on Ammonia nitrogen and volatile fatty acids (VFAs) in the rumen fluid of Naemi lambs.

Parameters %	Artificial Saliva (%)					SEM	*p Value*		
	0	1.5	3.0	4.5	6.0		*Treat*	*Linear*	*Quadratic*
Ammonia nitrogen	6.52	5.16	4.89	7.35	5.55	0.89	0.30	0.93	0.57
Acetic acid	21.63	22.37	26.69	32.63	21.16	3.71	0.20	0.46	0.15
Propionic acid	15.54	18.62	25.74	24.01	19.27	3.57	0.29	0.28	0.09
Iso-butyric acid	0.37	0.18	0.37	0.89	0.30	0.16	0.06	0.28	0.44
Butyric acid	2.66 ^c^	3.34 ^bc^	4.58 ^ab^	6.14 ^a^	4.25 ^b^	0.53	0.01	0.01	0.04
Iso-valeric acid	0.49 ^c^	0.91 ^c^	1.44 ^bc^	4.20 ^a^	1.76 ^b^	0.24	0.00	0.00	0.01
Valeric acid	0.55 ^b^	1.17 ^bc^	1.72 ^ab^	1.91 ^a^	0.46 ^c^	0.29	0.01	0.55	0.01
Total VFA	41.24 ^c^	46.58 ^bc^	60.53 ^ab^	69.7 ^a^	47.2 ^b^	7.07	0.05	0.15	0.04

^a, b, and c^ Means with different superscripts within each row were significantly different at *p* ≤ 0.05; SEM is the standard error of the mean; and *p* is the probability level.

## Data Availability

The original contributions presented in this study are included in the article. Further inquiries can be directed to the corresponding author(s).
